# Efficacy and safety of switching from Eculizumab to Ravulizumab for the maintenance of aHUS remission after kidney transplant: a preliminary experience

**DOI:** 10.1007/s40620-024-02005-7

**Published:** 2024-07-16

**Authors:** Marco Busutti, Federica Maritati, Greta Borelli, Claudia Bini, Valeria Corradetti, Vania Cuna, Chiara Abenavoli, Michele Provenzano, Matteo Ravaioli, Gaetano La Manna, Giorgia Comai

**Affiliations:** 1 Nephrology, Dialysis and Kidney Transplant Unit, IRCCS Azienda Ospedaliero-Universitaria Di Bologna, Via Massarenti 13, 40138 Bologna, Italy; 2 Department of Medical and Surgical Science (DIMEC), Alma Mater Studiorum University of Bologna, Bologna, Italy; 3 Hepato-Biliary Surgery and Transplant Unit, IRCCS Azienda Ospedaliero-Universitaria Di Bologna, Bologna, Italy

Ravulizumab (ULTOMIRIS™) is a C5-complement monoclonal antibody, engineered from eculizumab by the replacement of four amino-acids, leading to an increased duration of complement inhibition, with a more than fourfold longer half-life compared to its ancestor. Inhibitors of complement C5 have been developed for the treatment of atypical hemolytic uremic syndrome (aHUS) and paroxysmal nocturnal hemoglobinuria [1, 2]. Eculizumab has also proven effective in the treatment and prevention of aHUS relapses after kidney transplant (KT) [3, 4]. As ravulizumab is a long-acting version of eculizumab, we expect comparable efficacy and safety. Nevertheless, to date, data on the use of ravulizumab in KT recipients are scarce and need to be strengthened. The phase III trial ALXN1210-aHUS-311, designed to evaluate the efficacy and safety of ravulizumab in patients with aHUS, included only eight KT recipients (14.3% of enrolled patients) and their data were not evaluated separately from non-transplanted patients [5]. After ravulizumab approval, two cases have been reported regarding its use in KT patients. In the first, Schmidt et al. reported the effectiveness and safety of switching from eculizumab to ravulizumab for maintaining aHUS remission in a young living donor KT recipient [6]. In the second case, Jehn et al. describe the successful use of ravulizumab as induction and maintenance treatment in a patient with aHUS also after living donor KT [7].

Here, we report the cases of two young women who successfully switched from eculizumab to ravulizumab for aHUS remission maintenance after KT.

The first patient is a woman with aHUS complicated by end-stage kidney disease (ESKD), on eculizumab treatment since 2018. aHUS diagnosis was made after an episode of hemolytic macroangiopathic anemia and hypertensive crisis occurring after minor abdominal surgery. She had been on kidney replacement therapy for 2 years due to late referral for unknown nephropathy. Genetic testing revealed a rare heterozygous missense genetic variant (MAF 0.0002) in complement factor I (CFI) c.1386A > T(p.Gln462His), categorized as a variant of unknown significance (VUS), and the serum-induced C5b-9 deposition test on unstimulated and adenosine diphosphate (ADP)-activated human microvascular endothelial cells (HMEC-1) resulted positive, further confirming disease activity. In October 2021, the patient received a living donor ABO-incompatible (ABOi) KT. Induction therapy was based on basiliximab associated with rituximab and immunoadsorption. The maintenance immunosuppressive therapy consisted of tacrolimus, prednisone and azathioprine. At the time of KT, eculizumab administration was ongoing, no signs of hemolysis were detected and the C5b-9 deposition test had normalized. Thus, the drug was maintained in order to avoid aHUS relapse. At discharge, serum creatinine was 0.7 mg/dl and during the follow up graft function was stable without signs of hemolysis. In December 2022, because of difficulty in finding venous access eculizumab was replaced by ravulizumab according to the manufacturer’s indications. One year later, no relapse of aHUS has occurred. The patient has stable graft function and no proteinuria at urinalysis, nor have any adverse events been reported. The test of complement activation on HMEC-1 revealed no increase in C5b-9 deposits on either unstimulated or activated endothelium, indicating that adequate complement control had been achieved (Table 1).

**Table 1:** Laboratory characteristics, serum-induced C5b-9 deposition tests on inactivated and activated endothelieum results, functional assessment of chronic illness therapy—fatigue (FACIT-F) scores of the two patients at the time of switching from eculizumab to ravulizumab and during the first year after starting ravulizumab therapy

	M0 (switch)	Month 1	Month 6	Month 12
Patient 1				
Creatinine (mg/dl)	1.02	1.2	0.99	1.2
Hemoglobin (g/dl)	15.8	14.1	15.0	14.3
Platelets (× 10^9/mmc)	198	220	202	227
LDH (U/L)	234	206	191	203
UACR (mg/g)	31	18	21	40
C5b-9 deposition test—resting (normal values < 150%)	111%	–	105%	–
C5b-9 deposition test—activated (normal values < 150%)	134%	–	123%	–
FACIT-F	27	–	41	–
Patient 2				
Creatinine (mg/dl)	1.1	0.97	0.92	1.02
Hemoglobin (g/dl)	12.4	13.1	13.7	13.4
Platelets (× 10^9/mmc)	192	312	340	282
LDH (U/L)	183	174	160	148
UACR (mg/g)	–	25	5	4
C5b-9 deposition test—resting (normal values < 150%)	111%	–	98%	
C5b-9 deposition test—activated (normal values < 150%)	116%	–	124%	
FACIT-F	21	–	40	–

The second patient had a history of ESKD of unknown etiology, with a previous kidney transplant that lasted from 2004 to 2016. In 2016, a biopsy performed due to worsening graft function associated with hemolytic anemia, revealed a picture consistent with thrombotic microangiopathy associated with signs of chronic antibody-mediated rejection. These data were consistent with a diagnosis of active aHUS and the patient started eculizumab therapy. She also underwent complement genetic testing which identified a rare heterozygous genetic variant (MAF 0.0012) in the complement protein C3 (p.E484K), categorized as a VUS, together with other aHUS risk haplotypes (CFH-H3 and the MCP C.*897 T > C that tags the CD46ggaac haplotype). In addition, the in vitro test of complement activation on HMEC-1 revealed elevated C5b-9 deposits both on unstimulated and activated endothelium. In February 2022, she received a second kidney transplant from a living donor. Induction therapy consisted of basiliximab and maintenance therapy included tacrolimus, mycophenolate mofetil and prednisone. Eculizumab was continued after KT and the test of complement activation on HMEC-1 resulted normal. The post-operative course was characterized by immediate recovery of kidney function without signs of aHUS relapse, and the patient was discharged with a serum creatinine of 1.1 mg/dl. During the first year of follow up, graft function was stable and urinalysis within normal limits. In December 2022, due to concerns regarding the patient’s therapeutic compliance, we decided to switch from eculizumab to ravulizumab, according to the manufacturer’s instructions. One year later the drug is well tolerated, no aHUS relapse has been observed and graft function is stable (Table 1 and Fig. 1). In addition, the test of complement activation on HMEC-1 performed after three administrations of ravulizumab at nadir, that is 2 months after the previous infusion, remained normal.

In order to evaluate changes in quality of life, both patients were administered the Functional Assessment of Chronic Illness Therapy-Fatigue scale [8] questionnaire before, and 6 months after starting ravulizumab, which showed a remarkable improvement, especially in two of the four conceptual domains of quality of life, i.e., social well-being and emotional well-being (Table 1).

Our data suggest that switching C5 inhibition from eculizumab to ravulizumab to prevent aHUS recurrence in KT recipients may be effective, as was expected and consistently with other observations. Moreover, for the first time, the effectiveness of ravulizumab has been evaluated also using the serum-induced C5b-9 deposition test. This test, albeit not universally validated, is considered a sensitive tool for differentiating between active disease and remission and for monitoring eculizumab effectiveness in blocking the terminal complement pathway in aHUS.

Considering the limits related to small case series, Ravulizumab would appear to be safe and well tolerated, thus potentially leading to improvement in the patients’ quality of life as well as cost-effectiveness benefits, thus warranting further investigation.

## Data Availability

Data are available on request, reported in the hospital clinical records.
